# Supplementary material for: Long-read metagenomics of soil communities reveals phylum-specific secondary metabolite dynamics
Source: Commun Biol. 2021 Nov 18;4:1302. doi: 10.1038/s42003-021-02809-4 (PMC8602731; doi:10.1038/s42003-021-02809-4)
Supplement: Supplementary file 2 — Supplementary Information (new) [file 42003_2021_2809_MOESM2_ESM.pdf]

Supplementary Material for: "Long-read metagenomics of soil communities reveals phylum-specific secondary metabolite dynamics"

Marc W. Van Goethem[1], Andrew R. Osborn[1], Benjamin P. Bowen[1], Peter F. Andeer[1], Tami L. Swenson[1], Alicia Clum[2], Robert Riley[2], Guifen He[2], Maxim Koriabine[2], Laura Sandor[2], Mi Yan[2], Chris G. Daum[2], Yuko Yoshinaga[2], Thulani P. Makhalanyane[3], Ferran Garcia-Pichel[4,5], Ronan C. O'Malley[1,2], Axel Visel[2], Len A. Pennacchio[2], Trent R. Northen[1,2]*

[1] Molecular EcoSystems Biology Division, Lawrence Berkeley National Laboratory, 1 Cyclotron Rd, Berkeley, CA, 94720, USA

[2] DOE Joint Genome Institute, Lawrence Berkeley National Laboratory, 1 Cyclotron Rd, Berkeley, CA, 94720, USA

[3] Centre for Microbial Ecology and Genomics, Department of Biochemistry, Genomics and Microbiology, University of Pretoria, Lynnwood Rd, Hatfield. Pretoria, 0028, South Africa

[4] Center for Fundamental and Applied Microbiomics, Biodesign Institute, Arizona State University, Tempe, Arizona, USA

[5] School of Life Sciences, Arizona State University, Tempe, Arizona, USA

* Correspondence and requests for materials should be addressed to T.R.N. (email: TRNorthen@lbl.gov)

Prepared for: *Communications Biology*

Supplementary Note 1

Expression of Cyanobacterial Siderophore BGCs at Night

Differential gene expression analysis (DESeq2 ; $P < 0.05$; FDR = 5%) using mapped transcripts revealed that 10 BGCs contained biosynthetic genes that underwent significantly more transcription at night, all of which were cyanobacterial in origin. The most dramatic of these enrichments involved two cyanobacterial NRPS-PKS hybrid BGCs, identified on Node_81 and Node_86. These BGCs likely encode for a novel siderophore, putatively assigned based on genes encoding predicted membrane proteins involved in siderophore and iron transport located in the clusters (Fig. 2*c*). Additionally, a subset of cation acquisition genes was upregulated at night, suggesting a multifaceted approach for cation import at night that is under significant control of native regulatory constraints.

Node_81 (68 kb), appears to form a novel heptapeptide, while Node_86 (66 kb) is 2 kb shorter and has one fewer NRPS module, thus forming a hexapeptide (Fig. 2*c*). We speculated these to be rearranged BGCs based on the presence of transposases within Node_86, which were supported by differences in G+C content flanking the transposases, potentially indicating recent transposition. Overall, 80% of transposases located in BGCs were active across all time points. We were also able to recover BGCs from the assembled metatranscriptomic data (Supplementary Data 5).

Supplementary Note 2

Cation acquisition at night

To further investigate the transcriptional activity of cation acquisition genes at night, we mapped reads to all the co-assembled metagenomes. A subset of putative cation acquisition and sequestration gene were differentially expressed, specifically *hemH* (Ferrochelatase), *hxuB* (Heme/hemopexin transporter protein), *pacS* (putative copper-transporting ATPase) and *idiA* (iron ABC transporter) genes (Supplementary Data 6). The differential expression of siderophore

and cation acquisition genes at night suggests a common 'night-time' strategy of cation import in biocrust, which was consistent across most cyanobacterial BGCs (Figs. S6, S7).

Supplementary Note 3

Transposases are prevalent among biocrust BGCs

Two transposases and a phage integrase were found on Node_86, with the transposases located upstream of the final NRPS gene in the cluster. G+C content surrounding the transposases within the BGC had high levels of G-C skew (>1.5% deviation from the mean) potentially indicative of a recent gene rearrangement. We hypothesized that these mobile elements were responsible for the rearrangement of Node_81 into Node_86. This is supported by the perpetual expression of the two transposases and phage integrase in Node_86, implicating a mechanism for BGC rearrangement throughout the experiment and suggesting recombination events may still be occurring. We speculate that BGC re-arrangements may be on-going, long-term processes rather than brief, one-time events. Overall, 20% of transposases (Fig. S8) within BGCs were constitutively transcribed, hinting that similar long-term recombination events may be occurring in the biocrust community. Transposases located outside of BGCs, i.e., those more relevant to primary metabolism, showed less constitutive transcription (~7% of transposases) compared to those involved in secondary metabolism. Moreover, 26% of non-BGC transposases were never transcribed while only 19% of BGC transposases were never transcribed.

Nine of the 10 genes comprising Node_86 showed differential expression, with significantly higher transcription at night. As a putative siderophore, the function of this metabolite could be to acquire iron at night in preparation for photosynthesis the following day. In contrast, Node_81 only had one differentially expressed gene, but still tended towards nighttime activation.

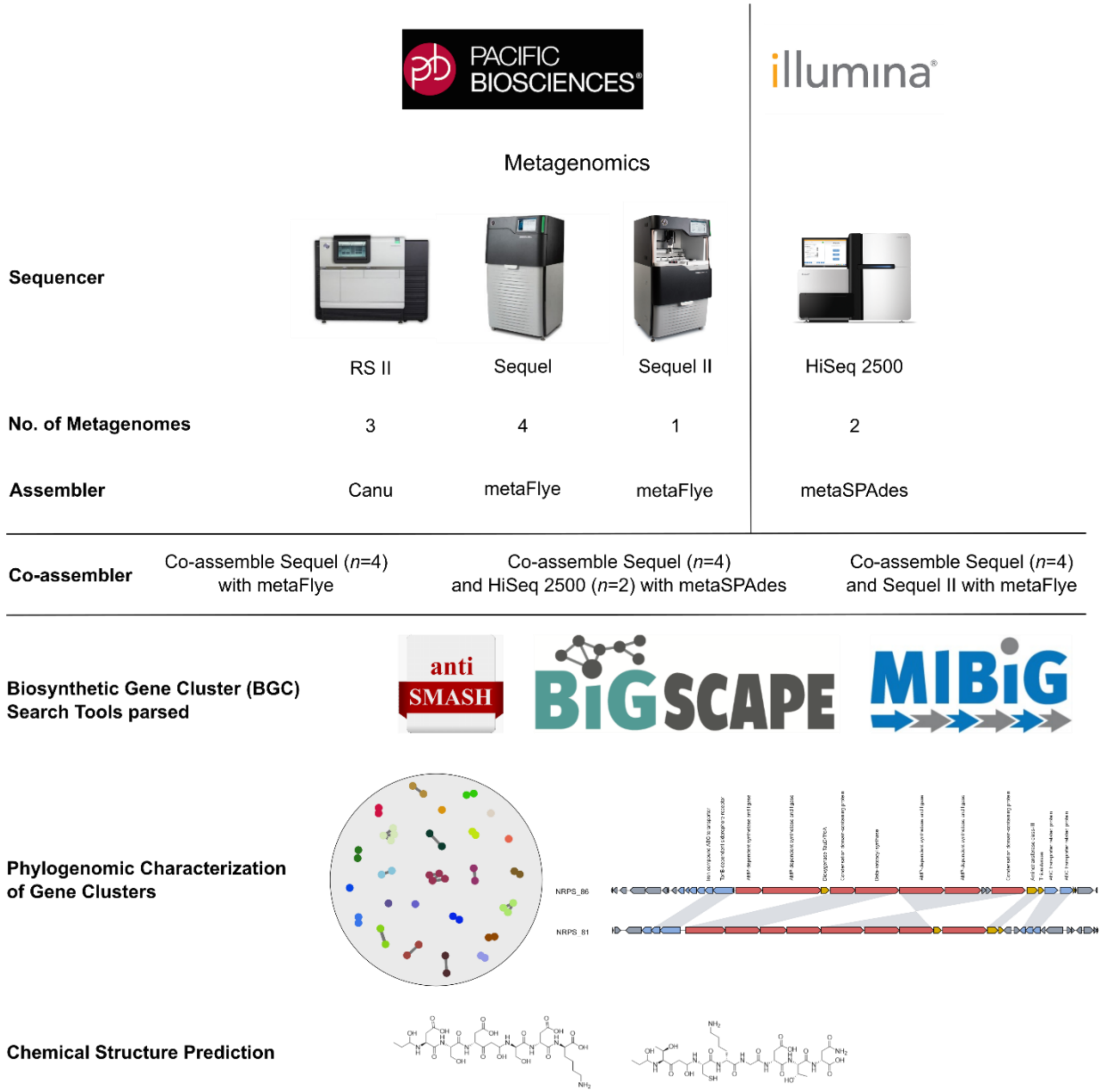

Supplementary Figure 1 | Workflow overview. 8 long-read metagenomes were generated using Pacific Biosciences instruments that span 3 generations (RS II -> Sequel -> Sequel II). 2 short-read metagenomes were generated from the same biocrust samples using an Illumina HiSeq 2500 instrument.

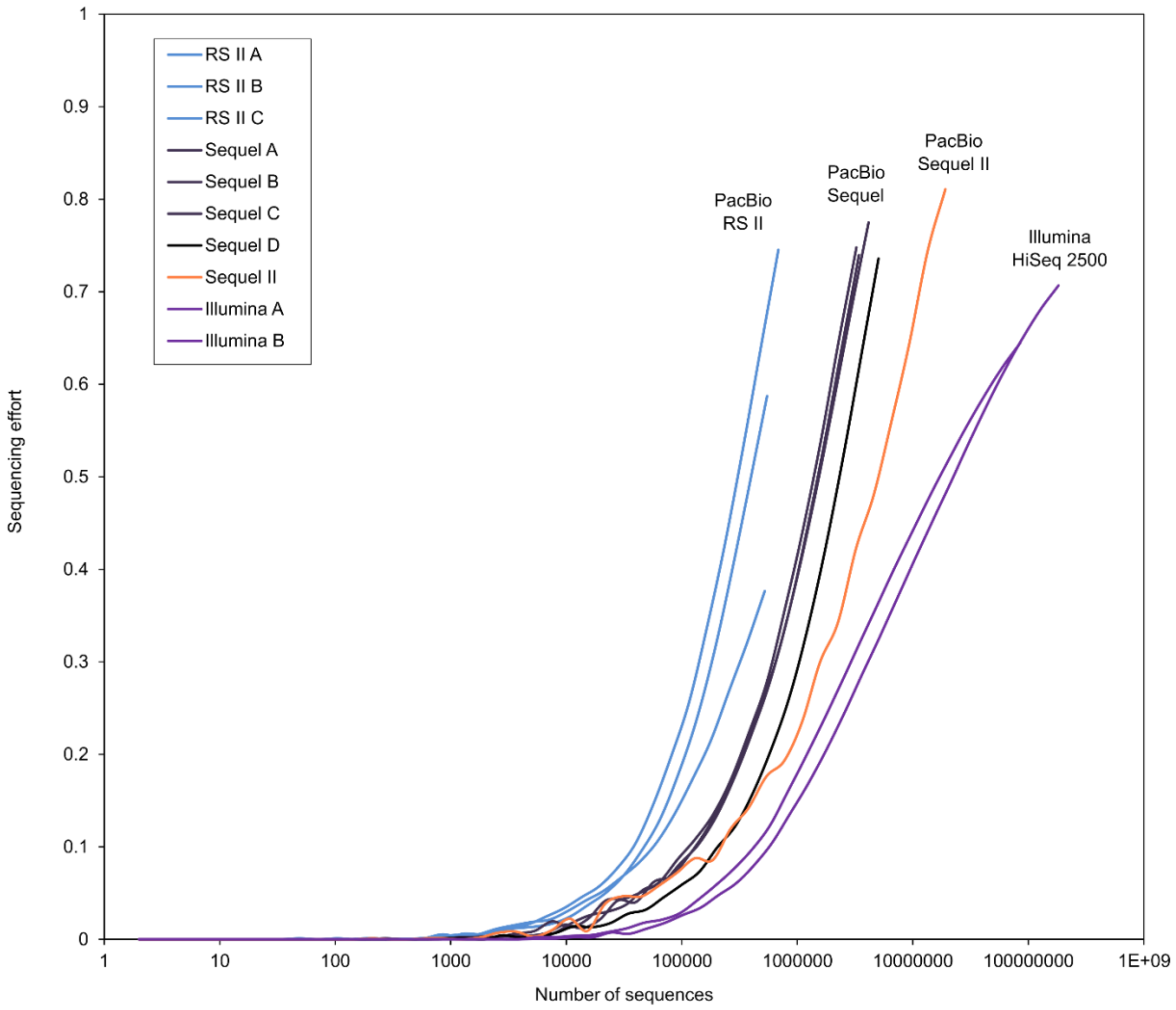

Supplementary Figure 2 | Estimates of sequencing effort. Nonpareil estimates sequence coverage by calculating read redundancy. The generations of long-read technology (RS II -> Sequel -> Sequel II) lead to improvements in sequencing depth. Short-read sequencing required orders of magnitude more sequencing to achieve a similar sequencing effort as RS II.

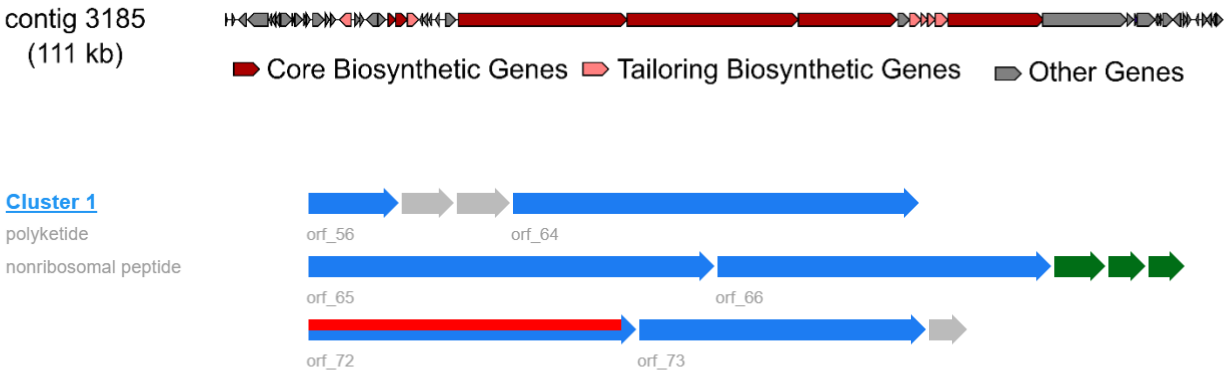

Supplementary Figure 3 | Longest BGC recovered from a metagenome. The longest BGC found across the metagenomes encodes a 111 kb transAT-PKS-NRPS. The domain architecture is provided by PRISM.

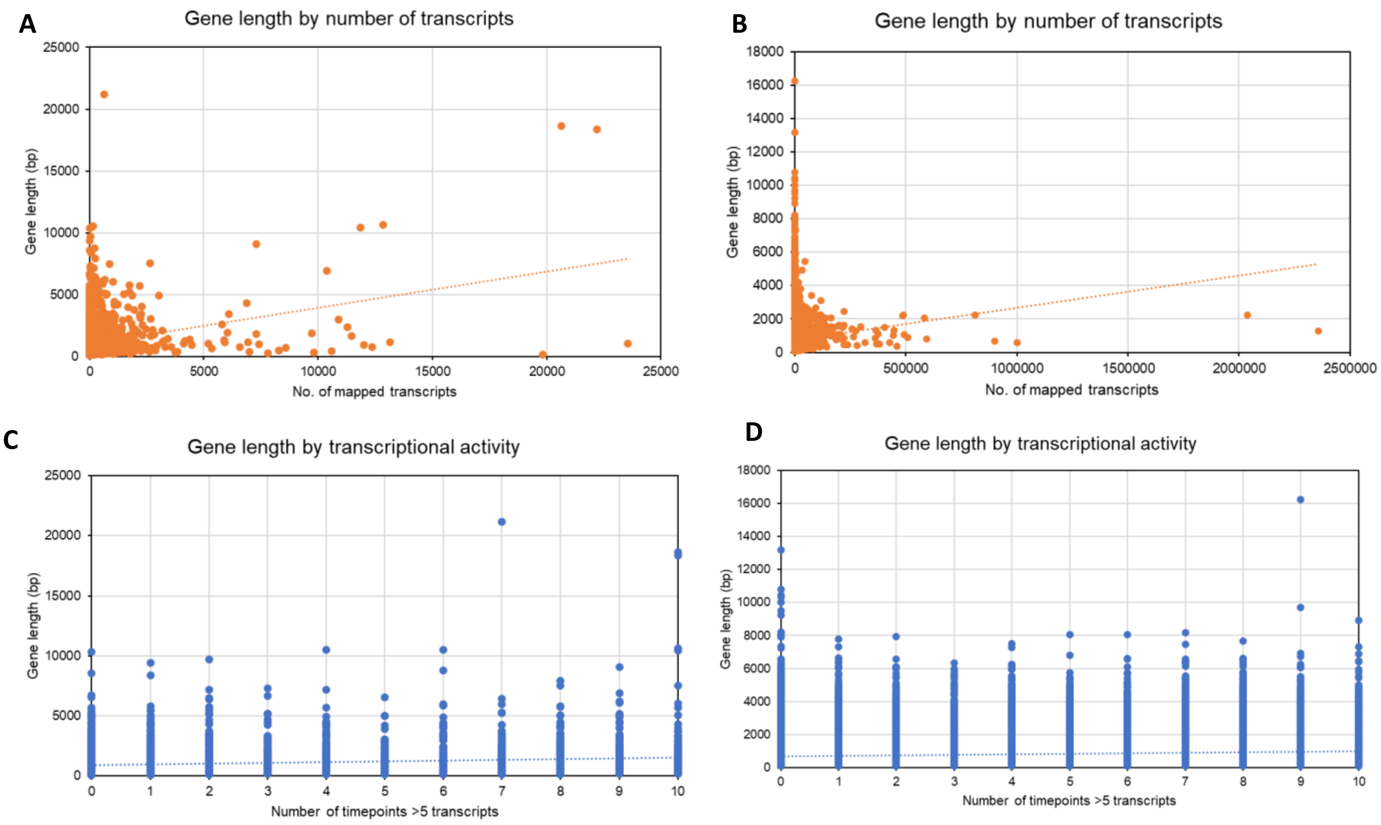

Supplementary Figure 4 | Mapping transcripts as a function of gene length. To test whether gene length influenced mapping rates we compared how read recruitment differed for the longer secondary metabolite genes (average gen e length=1153 bp) compared to the primary metabolic genes (average gene length=688 bp). Visualizing the number of mapped transcripts by gene length showed no correlation for either **a)** secondary or **b)** primary metabolic genes. Similarly, comparing the number of timepoints with 5 or more transcripts (where 0 means never expressed and 10 means constitutive expression) to gene length indicated no trend that followed an increase in transcript recruitment onto longer genes for either **c)** secondary or **d)** primary metabolic genes.

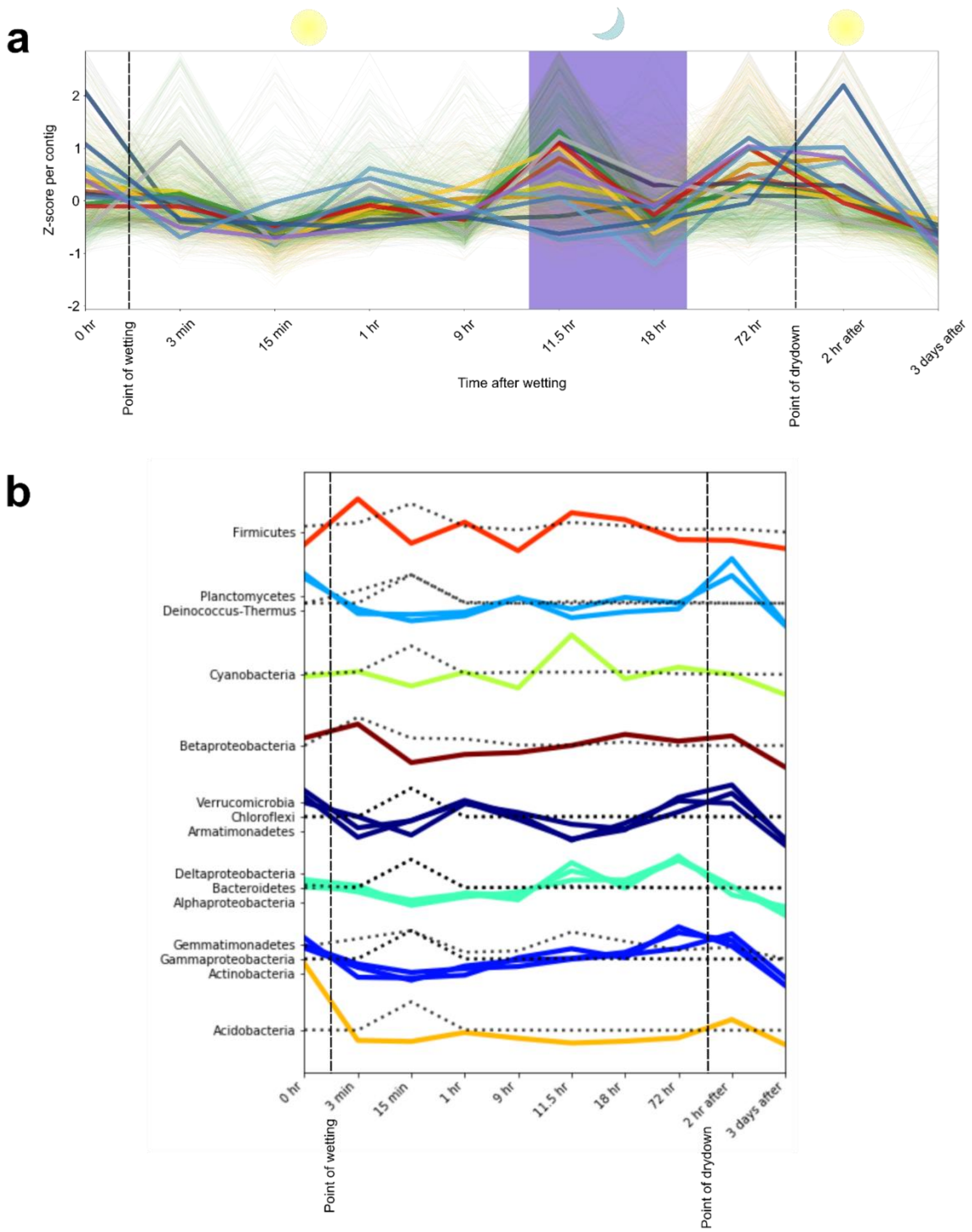

Supplementary Figure 5 | Diurnal trends in BGCs. a, Trends across the 3-day phase were detected in 12,470 expressed biosynthetic genes (cutoff > 20 mapped transcripts across time points). Read counts of transcript relative abundances per gene were Z-score normalized for the

purpose of visualization. Each gene trend is color-coded by its taxonomic affiliation. The purple background indicates night-time transcription. **b,** Clusters of bacterial phyla based on their average Z-score from all contigs with BGCs. The single-colored lines indicate secondary metabolism over time, while the dotted lines indicate the number of 16S rRNA transcripts at each timepoint.

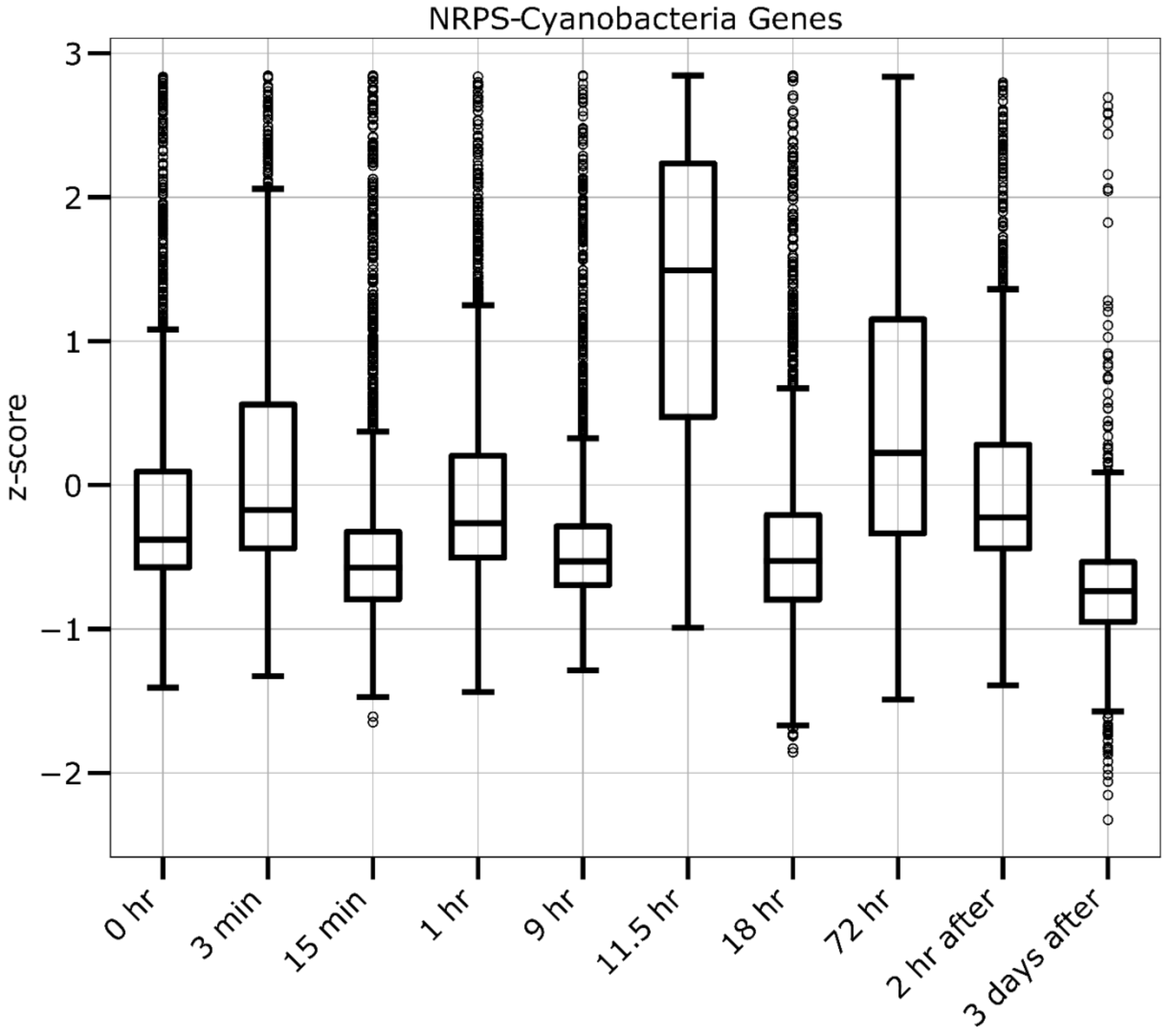

Supplementary Figure 6 | Cyanobacterial transcription occurs primarily at night. All NRPS gene clusters belonging to *Cyanobacteria* showed significantly more transcriptional activity 11.5 hours after wetting ($P < 0.05$). This first dark time point indicates a shunt of secondary metabolism by *Cyanobacteria* that also includes the increased transcription of RiPPs, T1PKS and T3PKS gene clusters. The boxes extend from Q1 to the Q3 quartile values of the data. The line is at the median (Q2) while the whiskers extend from the edges of the box (Q1 and Q3) to show the range of the data. The whiskers extend no more than 1.5 times the interquartile range (IQR = Q3 - Q1) from the edges of the box and end at the farthest data point within that interval. All outliers are plotted as separate dots beyond the whiskers.

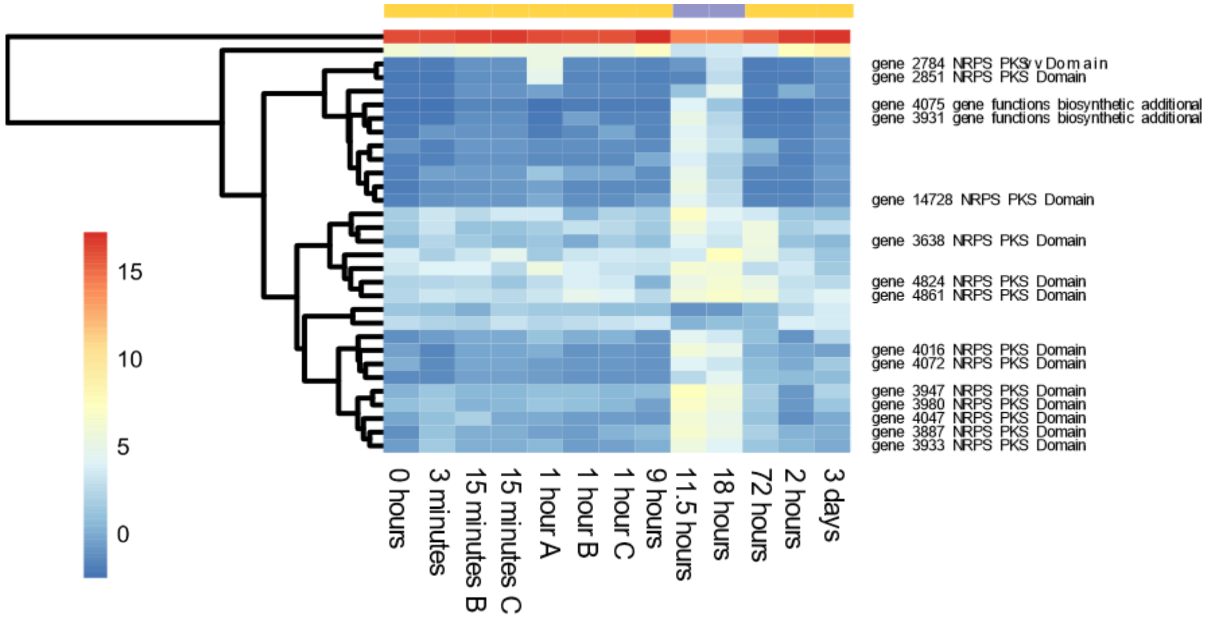

Supplementary Figure 7 | DESeq2 showed significantly enriched gene transcription at night. Genes labelled on the heatmaps were those located within BGCs (Flye co-assembly), while unlabeled rows are 'non-BGC' genes. Heatmap colors are based on DESeq2 comparisons between night and day based on Log2Fold changes. Higher Log2Fold changes are shown in warmer colors while cooler colors show less change between treatments. All genes are significantly differentially transcribed at night. Left color axis indicates the condition, i.e. day (yellow) or night (purple).

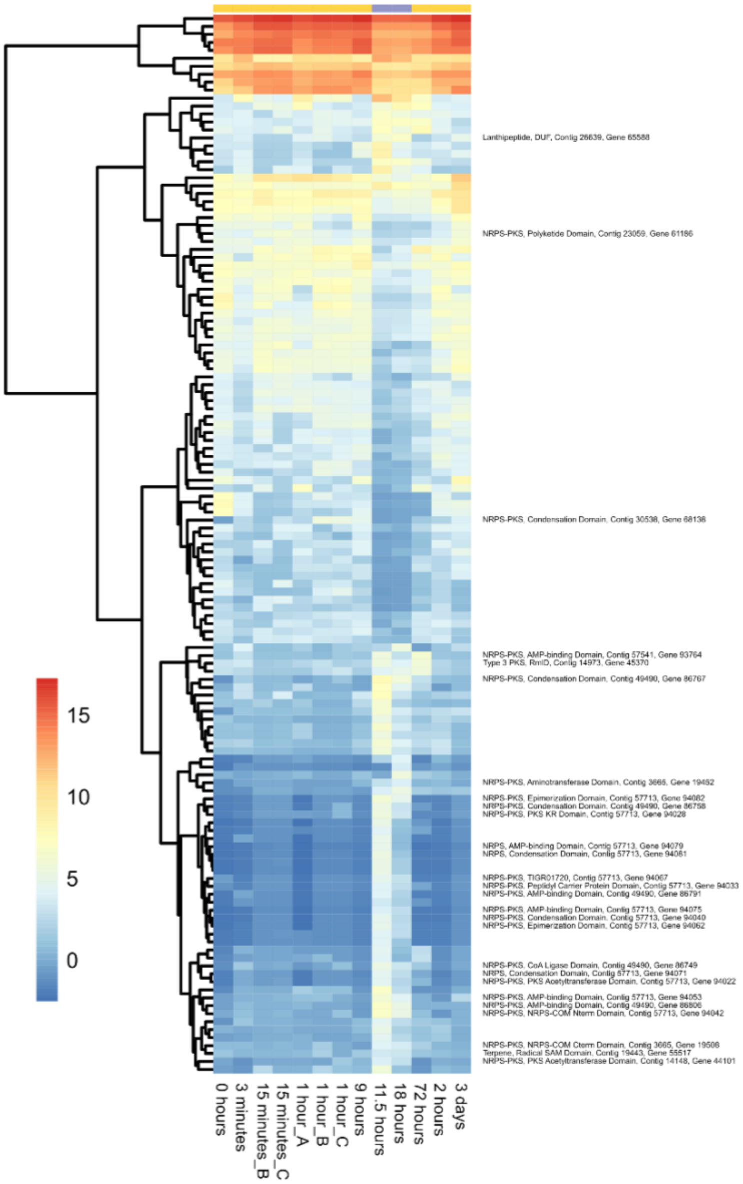

Supplementary Figure 8 | DESeq2 showed significantly enriched gene transcription at night. Genes labelled on the heatmaps were those located within BGCs (Ultimate co-assembly), while unlabeled rows are ‘non-BGC’ genes. Heatmap colors are based on DESeq2 comparisons between night and day based on Log2Fold changes. Higher Log2Fold changes are shown in warmer colors while cooler colors show less change between treatments. All genes are significantly differentially transcribed at night. Left color axis indicates the condition, i.e. day (yellow) or night (purple).

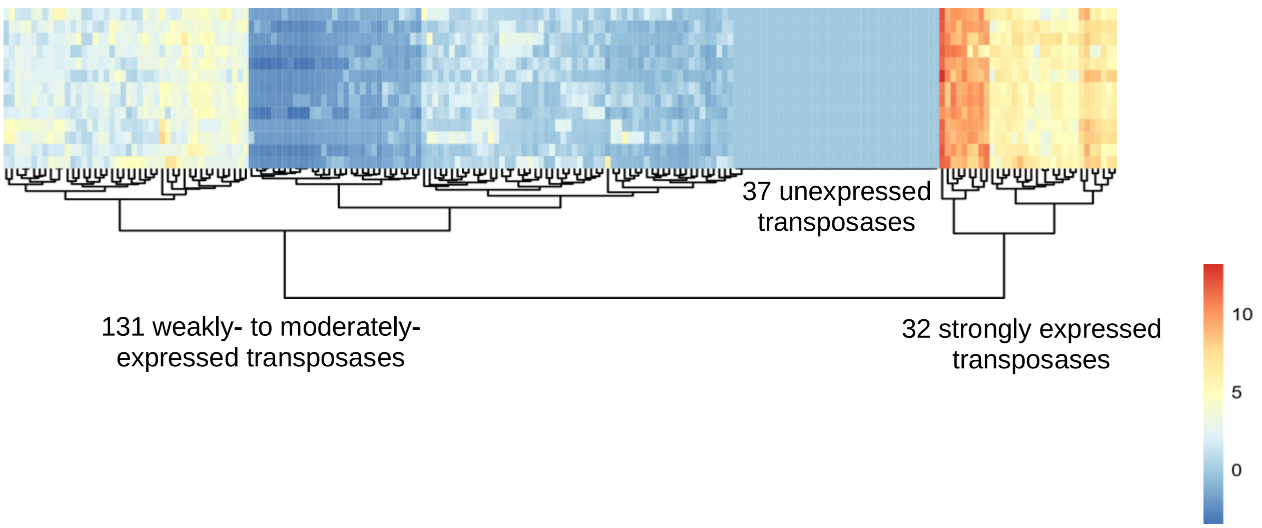

Supplementary Figure 9 | Transposase transcription. Heatmap showing the transcriptional Log2Fold change of all transposases located in BGCs over time, with 0 hours the bottom row and 3 days after wetting the top row as in Supplementary Figure 7. We identified 3 broad categories of expression: (i) unexpressed (no mapped transcripts), (ii) weak- to moderate-expression, and (iii) strongly expressed transposases. Reds indicate higher levels of transcription while blues are lowly-transcribed.

Supplementary References

1. Hiraoka, S. et al. Metaepigenomic analysis reveals the unexplored diversity of DNA methylation in an environmental prokaryotic community. *Nature communications* **10**, 159 (2019).
2. Frank, J.A. et al. Improved metagenome assemblies and taxonomic binning using long-read circular consensus sequence data. *Scientific reports* **6**, 25373 (2016).
3. Koren, S. et al. Canu: scalable and accurate long-read assembly via adaptive k-mer weighting and repeat separation. *Genome research* **27**, 722-736 (2017).
4. Nurk, S., Meleshko, D., Korobeynikov, A. & Pevzner, P.A. metaSPAdes: a new versatile metagenomic assembler. *Genome research* **27**, 824-834 (2017).
5. Kolmogorov, M., Rayko, M., Yuan, J., Polevikov, E. & Pevzner, P. metaFlye: scalable long-read metagenome assembly using repeat graphs. *bioRxiv*, 637637 (2019).
6. Mikheenko, A., Saveliev, V. & Gurevich, A. MetaQUAST: evaluation of metagenome assemblies. *Bioinformatics* **32**, 1088-1090 (2015).
7. Blin, K. et al. antiSMASH 5.0: updates to the secondary metabolite genome mining pipeline. *Nucleic acids research* (2019).
8. Love, M.I., Huber, W. & Anders, S. Moderated estimation of fold change and dispersion for RNA-seq data with DESeq2. *Genome biology* **15**, 550 (2014).
