# Supplementary material for: Long-read metagenomics of soil communities reveals phylum-specific secondary metabolite dynamics
Source: Commun Biol. 2021 Nov 18;4:1302. doi: 10.1038/s42003-021-02809-4 (PMC8602731; doi:10.1038/s42003-021-02809-4)
Supplement: Supplementary file 3 — Description of Additional Supplementary Files [file 42003_2021_2809_MOESM3_ESM.pdf]

Description of Additional Supplementary Files

File name: Supplementary Data 1
Description: Raw metagenome and metatranscriptome statistics.

File name: Supplementary Data 2
Description: Assembly statistics of short- and long-read metagenomes as well as metatranscriptomes.

File name: Supplementary Data 3
Description: Each biosynthetic gene cluster identified from the assembled metagenomes in this study.

File name: Supplementary Data 4
Description: Each biosynthetic gene cluster identified in the metatranscriptomic assemblies.

File name: Supplementary Data 5
Description: The genes used to calculate transcription of biosynthetic gene clusters and core bacterial genes.

File name: Supplementary Data 6
Description: DESeq2 analysis of significantly transcribed genes between day and night-time transcription.

File name: Supplementary Data 7
Description: Transcriptional scores for cation-related genes.

File name: Supplementary Data 8
Description: Average abundance pattern for each phylum through time.

File name: Supplementary Data 9
Description: Taxonomic composition of metagenomes and metatranscriptomes using full-length 16S rRNA.

File name: Supplementary Data 10
Description: Normalized sequence data showing scores of transcription at each time point with BGC type and Phylum shown
