# Supplementary material for: Long-read metagenomics of soil communities reveals phylum-specific secondary metabolite dynamics
Source: Commun Biol. 2021 Nov 18;4:1302. doi: 10.1038/s42003-021-02809-4 (PMC8602731; doi:10.1038/s42003-021-02809-4)
Supplement: Supplementary file 14 — Reporting Summary [file 42003_2021_2809_MOESM14_ESM.pdf]

nature portfolio

Corresponding author(s): Trent Northen

Last updated by author(s): Aug 31, 2021

Reporting Summary

Nature Portfolio wishes to improve the reproducibility of the work that we publish. This form provides structure for consistency and transparency in reporting. For further information on Nature Portfolio policies, see our Editorial Policies and the Editorial Policy Checklist.

Statistics

For all statistical analyses, confirm that the following items are present in the figure legend, table legend, main text, or Methods section.

n/a	Confirmed	
☐	☒	The exact sample size (*n*) for each experimental group/condition, given as a discrete number and unit of measurement
☐	☒	A statement on whether measurements were taken from distinct samples or whether the same sample was measured repeatedly
☐	☒	The statistical test(s) used AND whether they are one- or two-sided<br>*Only common tests should be described solely by name; describe more complex techniques in the Methods section.*
☒	☐	A description of all covariates tested
☐	☒	A description of any assumptions or corrections, such as tests of normality and adjustment for multiple comparisons
☐	☒	A full description of the statistical parameters including central tendency (e.g. means) or other basic estimates (e.g. regression coefficient) AND variation (e.g. standard deviation) or associated estimates of uncertainty (e.g. confidence intervals)
☐	☒	For null hypothesis testing, the test statistic (e.g. *F*, *t*, *r*) with confidence intervals, effect sizes, degrees of freedom and *P* value noted<br>*Give P values as exact values whenever suitable.*
☒	☐	For Bayesian analysis, information on the choice of priors and Markov chain Monte Carlo settings
☒	☐	For hierarchical and complex designs, identification of the appropriate level for tests and full reporting of outcomes
☐	☒	Estimates of effect sizes (e.g. Cohen's *d*, Pearson's *r*), indicating how they were calculated

Our web collection on statistics for biologists contains articles on many of the points above.

Software and code

Policy information about availability of computer code

Data collection	Metatranscriptomes sequenced from biocrust material collected at the same sampling site in Moab, Utah that were publicly-available on JGI GOLD (sequence project IDs 1010318 – 1022409). Additional sequence data from Lake Biwa were downloaded from the BioProject ID PRJDB6656. Additional sequence data from a biogas reactor were downloaded from BioProject PRJNA294734 with the accession numbers SRR212703,SRR2420276 and SRR2420280. The remaining sequence data were generated in this study as follows: We sequenced three SMRT cells on the PacBio RS II Single Molecule, Real-Time (SMRT®) DNA Sequencing System (Pacific Biosciences, CA, USA) using two different library inserts: 10 kb AMPure PB library [n=2] and a Low input 3 kb PB library [n=1] using binding kit P6 v2 with 360-minute and 120-minute movies for the respective libraries. The same libraries were then sequenced on a PacBio Sequel System (Pacific Biosciences) using Sequel Binding Kit 2.1 with a combination of 600- and 1200-minute movies. A third library was made using 10 kb AMPure PB approach with a Blue Pippin size cutoff of 4.5 kb. It was sequenced on PacBio Sequel II System (Pacific Biosciences) using 1.0 template prep kit and a 900-minute movie.
Data analysis	Sequence data were analyzed using nonpareil v3.30 to estimate sequence depth. 16S rRNA gene data were extracted from metagenomes using SortMeRNA v2.1b which were then analyzed using DADA2 with none default parameters used for truncation lenght (truncLen = 150) and maximum expected errors (maxEE = 1). Taxonomy of gene clusters was inferred by BLAST queries against the NCBI nr-database (hits retained when E-values < 1 X 10-10 and bit scores greater than 60). Long-read metagenomes were corrected and assembled using Canu v1.8 with an estimated mean genome size of 5 Mb (genomeSize=5m) and the following parameters as suggested by the authors of the software: corMinCoverage=0, corOutCoverage=all, corMhapSensitivity=high, correctedErrorRate=0.105, corMaxEvidenceCoverageLocal=10 and corMaxEvidenceCoverageGlobal=10. Long-read metagenomes were also assembled with metaFlye v2.4.2 with the --meta option implemented. Ilumina sequence data were quality controlled using Prinseq-lite v0.20.4 with --min_qual_mean set to 20 and -ns_max_n set to 0. Illumina assemblies and co-assemblies were produced using metaSPAdes v3.13.0. Open reading frames were predicted using Prodigal v2.6.3 and Prokka v1.14.6. Assembly quality was evaluated using MetaQUAST v5.0.2. Biosynthetic gene clusters (BGCs) were identified using antiSMASH v5.0 (online). BiG-SCAPE v0.0.0r and CORASON were used to evaluated gene similarities among BGCs. Duplicate contigs were removed with BB-Dedup. Long contigs with BGCs were taxonomically annotated using Contig Annotation Tool v5.0.4. Transcripts were mapped to contigs using bbmap v38.73. SAMtools v1.9 and Geneious were used to sort mapping files and to visualize mapping across contigs. DESeq2 v1.28.0 was used in R v3.6.3 to test gene transcription against the control time point (0 hours). Pearson two-sided pairwise

correlations were calculated on normalized values using Pingouin (v 0.4.0) Python package with a one-step Bonferroni correction. t-SNE (T-distributed Stochastic Neighbor Embedding) was used to visualize the biosynthetic gene transcription patterns in ordinance space with the sklearn (v 0.23.2) manifold module used with the following parameters: 'angle': 0.5, 'early_exaggeration': 12.0, 'init': 'random', 'learning_rate': 200.0, 'method': 'barnes_hut', 'metric': 'euclidean', 'min_grad_norm': 1e-07, 'n_components': 2, 'n_iter': 3000, 'n_iter_without_progress': 300, 'perplexity': 40, 'random_state': None, 'verbose': 1.

For manuscripts utilizing custom algorithms or software that are central to the research but not yet described in published literature, software must be made available to editors and reviewers. We strongly encourage code deposition in a community repository (e.g. GitHub). See the Nature Portfolio guidelines for submitting code & software for further information.

Data

Policy information about availability of data

All manuscripts must include a data availability statement. This statement should provide the following information, where applicable:

- Accession codes, unique identifiers, or web links for publicly available datasets
- A description of any restrictions on data availability
- For clinical datasets or third party data, please ensure that the statement adheres to our policy

Raw data of the long- and short-read biocrust metagenomes can be accessed on the IMG/M website (Submission ID 241874) or on the NCBI website (BioProject: PRJNA691698). The raw metatranscriptomic data are publicly-available through the JGI GOLD portal (sequence project IDs 1010318 – 1022409).

Field-specific reporting

Please select the one below that is the best fit for your research. If you are not sure, read the appropriate sections before making your selection.

☐ Life sciences ☐ Behavioural & social sciences ☒ Ecological, evolutionary & environmental sciences

For a reference copy of the document with all sections, see nature.com/documents/nr-reporting-summary-flat.pdf

Ecological, evolutionary & environmental sciences study design

All studies must disclose on these points even when the disclosure is negative.

Study description	Long- and short-read metagenomes were generated from biological soil crust material (biocrust) that had been in storage for ~2 months.
Research sample	An early-stage biological soil crust was aseptically collected from Moab, UT, USA in a petri dish. It was a representative sample of the type of biocrust in this region within the Green Butte Site (38°42’54.1’’N, 109°41’27.0’’W).
Sampling strategy	No sample sizes were determined as a single sample was used. This study was initially designed to develop long-read sequencing technologies on a complex environmental sample. However, the sample also forms part of larger sampling strategy for long-term ecological investigation in this area. As part of the associated study investigating community composition (bacteria and viruses) of biocrust, we collected 28 biocrust samples split equally among distinct stages of biocrust complexity (i.e. following a maturity gradient). These included replicates for early-stage biocrust, early-mid stage, late-mid stage and late-stage biocrust.
Data collection	*Describe the data collection procedure, including who recorded the data and how.*
Timing and spatial scale	*Indicate the start and stop dates of data collection, noting the frequency and periodicity of sampling and providing a rationale for these choices. If there is a gap between collection periods, state the dates for each sample cohort. Specify the spatial scale from which the data are taken*
Data exclusions	*If no data were excluded from the analyses, state so OR if data were excluded, describe the exclusions and the rationale behind them, indicating whether exclusion criteria were pre-established.*
Reproducibility	*Describe the measures taken to verify the reproducibility of experimental findings. For each experiment, note whether any attempts to repeat the experiment failed OR state that all attempts to repeat the experiment were successful.*
Randomization	*Describe how samples/organisms/participants were allocated into groups. If allocation was not random, describe how covariates were controlled. If this is not relevant to your study, explain why.*
Blinding	*Describe the extent of blinding used during data acquisition and analysis. If blinding was not possible, describe why OR explain why blinding was not relevant to your study.*

Did the study involve field work? ☐ Yes ☒ No

Reporting for specific materials, systems and methods

We require information from authors about some types of materials, experimental systems and methods used in many studies. Here, indicate whether each material, system or method listed is relevant to your study. If you are not sure if a list item applies to your research, read the appropriate section before selecting a response.

Materials & experimental systems

n/a	Involved in the study
☒	☐ Antibodies
☒	☐ Eukaryotic cell lines
☒	☐ Palaeontology and archaeology
☒	☐ Animals and other organisms
☒	☐ Human research participants
☒	☐ Clinical data
☒	☐ Dual use research of concern

Methods

n/a	Involved in the study
☒	☐ ChIP-seq
☒	☐ Flow cytometry
☒	☐ MRI-based neuroimaging
